# The pathogenic and clinical characteristics of community-acquired and hospital-acquired co-infection in patients hospitalized with severe fever with thrombocytopenia syndrome

**DOI:** 10.3389/fcimb.2026.1940837

**Published:** 2026-10-29

**Authors:** Cuiping Xia, Zhongle Cheng, Zhen Sun, Huijuan Bi, Jilu Shen

**Affiliations:** 1 Department of Clinical Laboratory, First Affiliated Hospital of Anhui Medical University, Hefei, China; 2 Department of Clinical Laboratory, Anhui Public Health Clinical Center, Hefei, China; 3 Department of Cardiology, First Affiliated Hospital of Anhui Medical University, Hefei, China

**Keywords:** community-acquired infections, epidemiology, risk factors, severe fever with thrombocytopenia syndrome, tick bite

## Abstract

**Background:**

Co-infection can accelerate the progression of SFTS, this study aims to analyze the clinical characteristics of SFTS-associated co-infection and identify the associated risk factors.

**Methods:**

In this retrospective study, we analyzed epidemiology, pathogen distribution patterns, and laboratory findings between community-acquired infections (CAIs) and hospital-acquired infections (HAIs) in SFTS patients. Our objectives were to determine the prevalence, characterize clinical manifestations, and identify potential risk factors associated with SFTS co-infection.

**Results:**

Among 231 SFTS patients, 81 (35.1%) had co-infection. SFTS patients with CAIs exhibited the highest mortality rate (23.4%). Compared to those without co-infection, patients with CAIs had more severe clinical presentation and poorer outcome. Antifungal treatment significantly improved survival in SFTS with CAIs-related fungal infections, but had no benefit in those with HAIs-related fungal infections. Pulmonary infections were associated with reduced survival time in SFTS patients with HAIs but had no significant impact on those with CAIs. Multivariate logistic regression analysis identified several independent risk factors for CAIs in SFTS patients, including diarrhea, cerebral infarction, antifungal therapy, ICU admission, elevated D-Dimer levels, and A/G <1.05. Among SFTS patients with co-infection, A/G <1.05 demonstrated potential predictive value for CAIs (AUC = 0.714, 95% CI: 0.626-0.832). Further analysis revealed that an A/G ratio <1.05 indicated moderate discriminatory performance for SFTS patients with CAIs related pulmonary fungal infections (AUC = 0.799, 95% CI: 0.702-0.896).

**Conclusions:**

SFTS patients with CAIs had poorer clinical outcomes than patients without co-infection or those with HAIs. An A/G ratio <1.05 may serve as a supportive marker for identifying patients at increased risk of CAIs and could assist clinical risk stratification.

## 1 Introduction

Severe fever with thrombocytopenia syndrome (SFTS) is a zoonotic infectious disease caused by a novel bunyavirus known as SFTSV. The disease typically has an acute onset and can progress to severe illness, particularly in immunocompromised individuals (Liu et al., 2013; Lei et al., 2015). SFTSV is primarily transmitted through tick bites. Following infection, the virus disseminates systemically and induces severe immune dysfunction. This triggers a cytokine storm and systemic inflammatory response, often culminating in multi-organ failure and high mortality (Wang et al., 2022). SFTS demonstrates distinct seasonal and geographic clustering, with reported mortality rates as high as 30% in endemic areas of East Asia (Kim et al., 2018; Ding et al., 2023).

SFTSV is frequently complicated by co-infection with other pathogens, including fungus and bacteria, which represent a significant prognostic factor that markedly reduces patient survival rate (Bae et al., 2020). The occurrence of co-infection in SFTS patients may be related to comorbidities, disease severity, and corticosteroid therapy (Liang et al., 2024). Notably, SFTS patients with pulmonary *Aspergillus* co-infection demonstrate worse clinical outcomes, including higher morbidity and mortality rates, compared to those without such co-infection (Zuo et al., 2023; Wang H. et al., 2024). Previous studies have reported an association between early antifungal therapy and improved survival in selected patients with pulmonary aspergillosis. Currently, as no specific antiviral treatment exists for SFTS, management remains limited to supportive care (Park et al., 2016; Zhang et al., 2023). Given the rapid progression of SFTS, prompt identification and management of risk factors are crucial for optimizing treatment strategies, improving patient outcomes, and reducing mortality.

Current understanding of SFTS remains limited, especially concerning co-infection types, pathogen distribution patterns, and clinical outcomes associated with both community-acquired infections (CAIs) and hospital-acquired infections (HAIs). Elucidating the clinical characteristics and predictors for these co-infection is essential for guiding appropriate antibiotic selection in SFTS patients. Therefore, this retrospective study aims to: (1) determine the incidence rate of CAIs and HAIs among SFTS patients, (2) characterize the distribution patterns and clinical outcomes of bacterial and fungal co-infection, and (3) analyze the epidemiological features and laboratory parameter differences between CAIs and HAIs. Identification of potential risk factors may contribute to the development of future risk-assessment strategies.

## 2 Materials and methods

### 2.1 Study design

This retrospective study was conducted at the First Affiliated Hospital of Anhui Medical University, utilizing data collected from May 2021 to June 2024. A confirmed diagnosis of an acute SFTS patient required a positive result of SFTSV RNA or specific IgM antibody test upon admission. Suspected cases were defined as patients who met the following criteria: (1) history of outdoor work, travel during peak incidence seasons, or tick bite exposure within 2 weeks prior to symptom onset; and (2) presentation with acute-onset high fever (temperature > 38°C), accompanied by laboratory findings of leukopenia, thrombocytopenia, and elevated liver transaminase (Yoshikawa et al., 2014). The study included 231 confirmed and suspected SFTS cases. This study has obtained written consent from the Ethics Committee of the First Affiliated Hospital of Anhui Medical University (Ethics Approval Number: PJ-YX2025-040), and were introduced according to the principles of the Declaration of Helsinki. Given the retrospective nature of our study, therefore, an application for exemption of informed consent was submitted.

Based on clinical manifestations, we classify SFTS patients with co-infection into mild, moderate, severe, and critically ill cases (Bae et al., 2022). Mild cases may exhibit slight laboratory changes and clinical manifestations similar to influenza, while moderate cases may present with fever, bleeding, gastrointestinal and neurological symptoms, as well as decreased platelets and white blood cells. In addition to the above symptoms, severe and critically ill patients may also exhibit multi organ dysfunction, microcirculation disorders, and even shock.

### 2.2 Data collection and definitions

Clinical data were extracted from the hospital’s electronic medical records, including demographic data, epidemiological characteristics, underlying comorbidities, clinical symptoms and signs on admission, laboratory test results, treatment regimens and clinical outcomes.

SFTS with co-infection is defined as the emergence of new pathogenic positive evidence in SFTS patients after admission. Clinically, pathogen detection can be performed through one or more methods after the patient’s admission such as microbial culture, Metagenomic next-generation sequencing (mNGS), IgM antibody test, Fungal fluorescence staining and Gram staining, (1,3)-β-D-Galactomannan test (G test) and galactomannan test (GM test), *Aspergillus* IgG antibody quantification. Even if no pathogen is detected, combined with SFTS patient’s clinical symptoms and imaging examinations (such as lung consolidation, ground-glass shadow, cavities, etc.), it can still be diagnosed as co-infection. All of the 231 SFTS patients in this study were tested at least once during their hospitalization for microbiological examination. This study excluded SFTS patients who had other pathogenic microbial infections before SFTS or used antibiotic treatment. According to the Centers for Disease Control and Prevention (CDC), SFTS patients with CAIs is defined as: (i) Within 48 hours after admission, any body part tests positive for pathogens; (ii) No hospitalization, surgery, or long-term care institution admission history within the past year; (iii) Currently, there are no indwelling percutaneous devices or indwelling catheters (Minnesota Department of Health, 2004). SFTS patients with HAIs is defined as the acquisition of infectious agent(s) which occurs after 48 hours or more of hospital admission, and there must be no above evidence that the co-infection was present or incubating at the time of admission to the acute care setting (CDC, 1989; Horan et al., 2008).

### 2.3 Statistical analysis

Statistical analyses were performed using SPSS 24.0 (IBM Corp., Armonk, NY). Continuous variables were expressed as mean ± SD or median (95% confidence intervals, 95% CI), and comparisons were made using Student t or Mann-Whitney U test as appropriate based on the normal distribution characteristics of the variables. Categorical variables were presented as frequencies and percentages as n (%), and analyzed using Pearson or Fisher’s exact chi-square test. Prior to conducting multivariable logistic regression, Lasso regression analysis was performed to address multicollinearity among variables and identify independent risk factors for SFTS with co-infection. The predictive performance of these risk factors was assessed using receiver operating characteristic (ROC) curve analysis, with sensitivity and specificity reported. Survival times between groups were compared using Kaplan-Meier analysis with log-rank testing. Correlations between the white blood cell ratio and other hematological parameters were evaluated using Pearson or Spearman correlation analysis, depending on data distribution. A two-tailed *P* < 0.05 was considered statistically significant.

## 3 Results

### 3.1 Clinical characteristics of patients with SFTS

This study included 231 SFTS cases, comprising 95 male (41.1%) and 136 female (58.9%) patients, with a mean age of 65.0 ± 11.7 years (**Table 1**). 150 cases (64.9%) had no co-infection, while 81 (35.1%) presented with co-infection, including 47 cases (20.3%) of CAIs and 34 cases (14.7%) of HAIs. 57 cases (24.7%) had a history of tick bites upon admission, with no significant epidemiological differences observed between SFTS patients with or without co-infection (*p*>0.05) (**Table 1**).

**Table 1:** Clinical characteristics of SFTS patients.

Variables	Total cohort (N=231)	Non-co-infection (n=150)	CAIs (n=47)	HAIs (n=34)	*P* ^a^ Value	*P* ^b^ Value
Demographics, n (%)						
Gender					0.282	0.106
Female	136 (58.9)	89 (59.3)	32 (68.1)	15 (44.1)		
Male	95 (41.1)	61 (40.7)	15 (31.9)	19 (55.9)		
Age (years)	65.04 ± 11.71	63.87 ± 12.48	66.80 ± 11.19	67.79 ± 7.67	0.285	0.294
Time from onset to admission (days)	5.00 (5.15-6.05)	5.00 (4.94-5.67)	6.00 (4.87-7.93)	5.00 (4.09-7.49)	0.281	0.644
Vital signs, n (%)						
Heart rate (times/minute)	85.00 (83.00-87.22)	85.00 (82.30-87.30)	85.00 (82.85-94.17)	81.00 (76.71-86.99)	0.422	0.248
Breath (times/minute)	20.00 (20.22-21.62)	20.00 (19.96-20.97)	20.00 (19.86-25.92)	20.00 (19.40-21.02)	0.184	0.963
Body temperature (°C)	37.50 (37.39-37.64)	37.55 (37.35-37.67)	37.60 (37.35-37.88)	37.40 (37.09-37.70)	0.610	0.464
Systolic pressure (mmHg)	115.00 (113.93-118.70)	114.50 (113.14-118.99)	115.00 (112.69-124.62)	117.00 (108.25-120.18)	0.533	0.550
Diastolic pressure (mmHg)	71.50 (71.00-74.13)	71.50 (71.41-75.17)	72.00 (68.09-76.35)	71.00 (66.06-73.64)	0.423	0.170
Past history, n (%)						
Bite by ticks	57 (24.7)	42 (28.0)	7 (14.9)	8 (23.5)	0.070	0.597
History of smoking	16 (6.9)	10 (6.7)	2 (4.3)	4 (11.8)	0.735	0.296
History of alcohol	14 (6.1)	9 (6.0)	1 (2.1)	4 (11.8)	0.457	0.264
Diabetes mellitus	35 (15.2)	22 (14.7)	7 (14.9)	6 (17.6)	0.969	0.662
Hypertension	82 (35.5)	51 (34.0)	19 (40.4)	12 (35.3)	0.422	0.886
Coronary heart disease	13 (5.6)	10 (6.7)	2 (4.3)	1 (2.9)	0.735	0.692
Cerebral infarction	60 (26.0)	27 (18.0)	23 (48.9)	10 (29.4)	0.000	0.134
Initial symptoms, n (%)						
Chest distress	15 (6.5)	11 (7.3)	3 (6.4)	1 (2.9)	1.000	0.699
Chest pain	10 (4.3)	6 (4.0)	2 (4.3)	2 (5.9)	1.000	0.642
Diarrhea	68 (29.4)	34 (22.7)	27 (57.4)	7 (20.6)	0.000	0.793
Sickness	38 (16.5)	29 (19.3)	6 (12.8)	3 (8.8)	0.304	0.144
Abdominal distension	6 (2.6)	6 (4.0)	0 (0.0)	0 (0.0)	0.339	0.595
Muscle soreness	65 (28.1)	38 (25.3)	13 (27.7)	14 (41.2)	0.751	0.064
Cough	35 (15.2)	25 (16.7)	7 (14.9)	3 (8.8)	0.774	0.25
Expectoration	43 (18.6)	29 (19.3)	8 (17.0)	6 (17.6)	0.723	0.821
Skin redness and swelling	17 (7.4)	14 (9.3)	2 (4.3)	1 (2.9)	0.367	0.311
Weak	113 (48.9)	74 (49.3)	26 (55.3)	13 (38.2)	0.474	0.242
Dizziness	74 (32.0)	44 (29.3)	18 (38.3)	11 (32.4)	0.248	0.728
Cephalalgia	20 (8.7)	9 (6.0)	5 (10.6)	6 (17.6)	0.329	0.037
Consciousness disorder	20 (8.7)	12 (8.0)	6 (12.8)	2 (5.9)	0.383	1.000
Drowsiness	8 (3.5)	6 (4.0)	2 (4.3)	0 (0.0)	1.000	0.595
Hemorrhage	47 (20.3)	28 (18.7)	12 (25.5)	7 (20.6)	0.307	0.797
Clinical therapy, n (%)						
Ribavirin	139 (60.2)	79 (52.7)	36 (76.6)	24 (70.6)	0.004	0.057
Immunoglobulins	195 (84.4)	124 (82.7)	40 (85.1)	31 (91.2)	0.696	0.219
Antibiotic therapy	207 (89.6)	130 (86.7)	47 (100)	30 (88.2)	0.005	1.000
Antifungal therapy	38 (16.5)	3 (2.0)	23 (48.9)	12 (35.3)	0.000	0.000
Blood Products	130 (56.3)	73 (48.7)	33 (70.2)	24 (70.6)	0.010	0.021
Glucocorticoids	108 (46.8)	72 (48.0)	21 (44.7)	15 (44.1)	0.691	0.682
Oxygen-inspiration	118 (51.1)	71 (47.3)	28 (59.6)	19 (55.9)	0.143	0.368
Endotracheal intubation	34 (14.7)	20 (13.3)	10 (21.3)	4 (11.8)	0.186	1.000
Transfer to ICU	17 (7.4)	4 (2.7)	9 (19.1)	4 (11.8)	0.000	0.040
Complication, n (%)						
Sepsis	17 (7.4)	10 (6.7)	5 (10.6)	2 (5.9)	0.358	1.000
Viral Encephalitis	15 (6.5)	8 (5.3)	3 (6.4)	4 (11.8)	0.726	0.239
Rhabdomyolysis	11 (4.8)	8 (5.3)	3 (6.4)	0 (0)	0.726	0.355
Myocardial injury	10 (4.3)	3 (2.0)	6 (12.8)	1 (2.9)	0.007	0.562
Respiratory failure	17 (7.4)	7 (4.7)	7 (14.9)	3 (8.8)	0.044	0.396
Clinical type, n (%)					0.000	0.003
mild/moderate cases	95 (41.1)	78 (52.0)	9 (19.1)	8 (23.5)		
severe/critically ill cases	136 (58.9)	72 (48.0)	38 (80.9)	26 (76.5)		
Outcome, n (%)						
Survival	188 (81.4)	121 (80.7)	36 (76.6)	31 (91.2)	0.545	0.144
Death	43 (18.6)	29 (19.3)	11 (23.4)	3 (8.8)		
Hospitalization stay (days)	9.00 (9.59-12.24)	8.00 (7.48-9.46)	11.00 (8.92-18.90)	16.00 (14.60-20.67)	0.015	0.000
Hospitalization expenses (CNY¥)	31818 (34877-47995)	27597 (25369-35037)	44006 (36727-76139)	48813 (44045-97314)	0.000	0.000

CAIs, Community-acquired infections; HAIs, hospital-acquired infections; ICU, Intensive care unit; CNY, Chinese Yuan.

^a^ Comparison between non-co-infection and CAIs; ^b^ Comparison between non-co-infection and HAIs. Statistical analysis for continuous variables was performed using Student’s t-test or Mann-Whitney U test, while categorical variables were analyzed using Pearson’s chi-squared test or Fisher’s exact test; the denominator of the percentages displayed in **Table 1** was the total number (n) corresponding to the respective group denominators.

The most frequent clinical manifestations among SFTS patients included weak (113, 48.9%), dizziness (74, 32%), diarrhea (68, 29.4%), and muscle soreness (65, 28.1%). CAIs patients exhibited a significantly higher incidence of diarrhea compared to non-coinfected patients (57.4% vs 22.7%). HAIs patients reported cephalalgia more frequently than other groups (*p* < 0.05). CAIs patients were more likely to have a history of cerebral infarction than those without co-infection (*p* < 0.000). No significant differences in comorbidities were observed between HAIs patients and non-coinfected individuals (*p*>0.05).

The hematological test results upon admission showed that compared to SFTS patients without co-infection, CAIs patients had cTnI (0.06ng/L vs 0.04ng/L, *p* = 0.007), AMY (102.00U/L vs 91.50U/L, *p* = 0.028), LPS (600.00U/L vs 406.00U/L, *p* = 0.026), D-D (3.41 μ g/mL vs 2.15 μg/mL, *p* = 0.006) and FDP (11.26 μg/mL vs 7.38 μg/mL, *p* = 0.031) significantly increased, while the ALB and A/G ratios significantly decreased. In clinical practice, elevated cTnI levels are the “gold standard” biomarker for myocardial injury, the combination of AMY and LPS testing is the preferred combination for screening acute pancreatitis, and elevated levels of D-D and FDP reflect an imbalance between the body’s coagulation and fibrinolysis systems, commonly observed in pathological hyperfibrinolytic states. These results indicated that CAIs patients exhibited more severe myocardial injury, pancreatic injury, and coagulation dysfunction. There was no significant difference in hematological parameters between HAIs and SFTS patients without co infection (*p*>0.05).meter.

The clinical interventions for SFTS patients should be determined based on a comprehensive assessment of disease severity, risk of complications, and individual differences. In this study, compared to SFTS patients without co-infection, those with CAIs were significantly more likely to receive ribavirin antiviral treatment, antibiotics, antifungal treatment, and blood product transfusions (*p* < 0.05) (**Table 1**). While HAI patients showed no difference in ribavirin and antibiotic use compared to non-coinfected patients (*p*>0.05), they resembled CAIs patients in their higher likelihood of receiving antifungal and blood product (*p* < 0.05). Additionally, both CAIs and HAIs patients had higher ICU admission rates. No significant differences were observed in glucocorticoid use among CAIs and HAIs and non-coinfected patients (*p*>0.05).

Compared with SFTS patients without co-infection (**Table 1**), patients with CAIs had poorer clinical outcomes, longer hospital stays, higher hospitalization costs, and a higher incidence of myocardial injury and respiratory failure (*p* < 0.05). The mortality rate was also higher among patients with CAIs (23.4%, 11/47) than among patients with HAIs (8.8%, 3/34) or those without co-infection (19.3%, 29/150). In contrast, patients with HAIs did not show significant differences in clinical outcomes compared with patients without co-infection (*p*>0.05).

### 3.2 Laboratory evidence of co-infection in hospitalized SFTS patients

Among CAIs-related bacterial infections, Gram staining of sputum smear identified pathogens in 7 patients, while chest CT findings suggested pulmonary infection in 2 additional cases. All fungal infections associated with CAIs were confirmed through culture methods. For HAIs-related bacterial infections, 3 case was diagnosed via sputum smear gram staining, with chest CT indicating pulmonary infection in 9 patients. Regarding HAIs-related fungal infections, 2 cases were diagnosed using (1,3)-β-D-Galactomannan testing, while chest CT revealed pulmonary infection in 7 patients (**Table 2**).

**Table 2:** Laboratory detection methods for SFTS patients with co-infection.

	Co-infection (N)	
Characteristic	CAIs	HAIs
Bacteria (n,%)		
Culture	25	12
Gram staining	7	3
mNGS	7	10
CT evidence	0	2
IgM antibody	2	2
Fungus (n,%)		
Culture	29	9
mNGS	0	5
Galactomannan/1-3-β-D glucan test	0	2
CT evidence	2	7
Virus (n,%)		
mNGS	1	1

CAIs, Community-acquired infections; HAIs, Hospital-associated infections; mNGS, Metagenomic next-generation sequencing; CT, computerized tomography.

Among SFTS patients with confirmed co-infection, 40 cases were classified as CAIs who detected specific pathogens (**Figure 1C**) (**Supplementary Table 1**). Pathogen identification included 54 pathogens by culture method, 7 pathogens by mNGS, and 2 pathogens by IgM antibody quantification; 23 cases were HAIs who detected specific pathogens (**Figure 1D**) (**Supplementary Table 1**), among which 21 pathogens by the culture method, 15 pathogens by mNGS, and 2 pathogens by IgM antibody quantification (**Table 2**).

**Figure 1:**
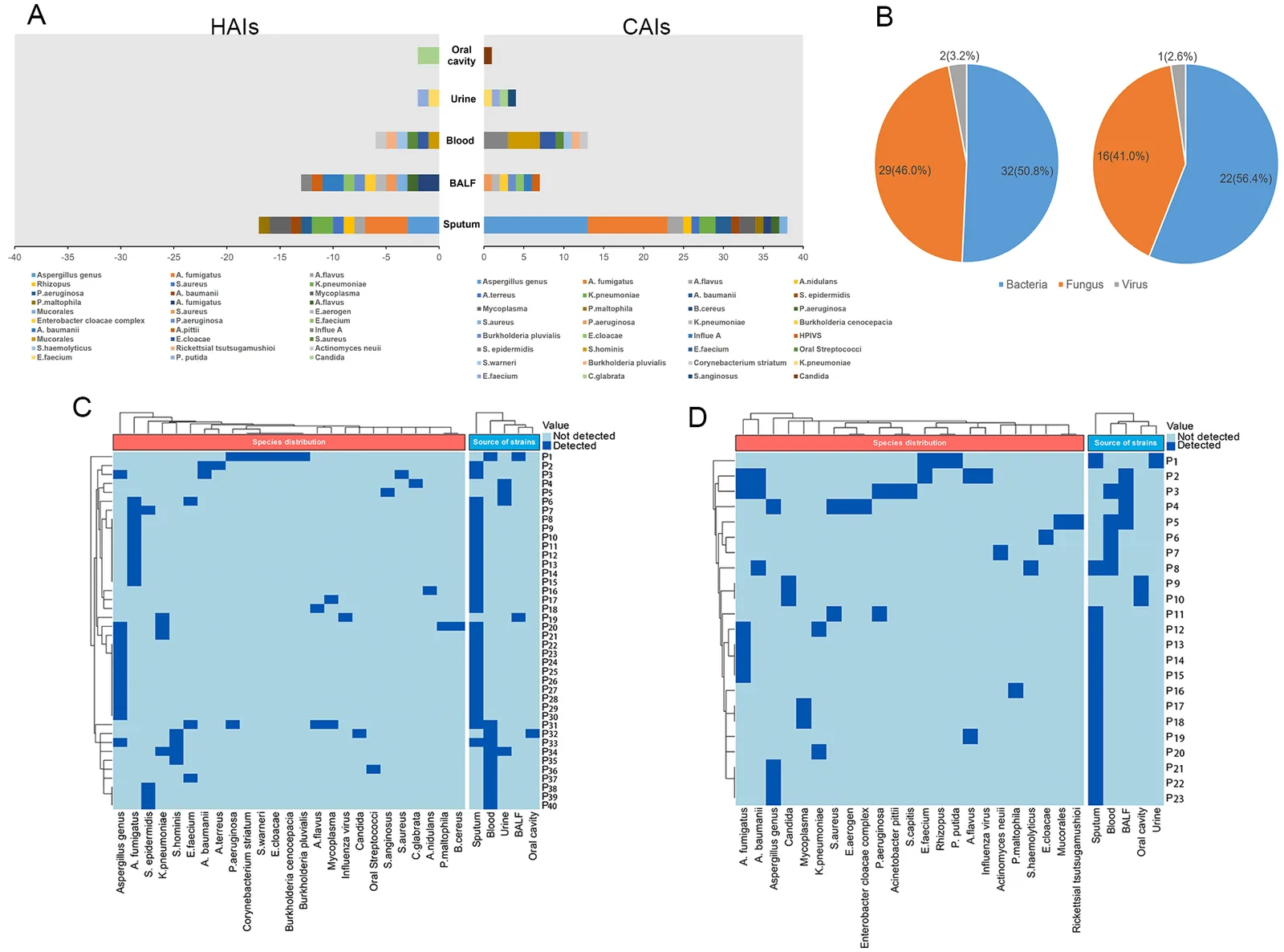
Pathogen distribution in SFTS patients with co-infection. **(A)** Sample sources of pathogens isolated from SFTS patients with CAIs and HAIs. **(B)** Relative abundances of bacterial, fungal, and viral co-infection in SFTS patients. **(C)** Pathogen distribution detected in SFTS patients with CAIs. **(D)** Pathogen distribution detected in SFTS patients with HAIs.

### 3.3 Pathogen types and distribution in co-infection

In SFTS with CAIs, we detected 63 pathogens. The most prevalent were *Aspergillus genus* (13/63), followed by *A. fumigatus* (10/63), *S.hominis* (4/63), *K.pneumoniae* (4/63), *S. epidermidis* (4/63) (**Table 3**). Pathogen distribution by sample type was as follows: sputum (38), blood (13), BALF (6), urine (4), and oral secretions (2). Among SFTS patients with HAIs, we identified 39 pathogens, predominantly *A. fumigatus* (6/39), followed by *Aspergillus genus* (3/39), *A. baumanii* (3/39), *S.aureus* (2/39), *A.flavus* (2/39). The distribution across sample types showed: sputum (17), BALF (13), blood (6), with urine and oral secretions each containing 2 pathogens. Notably, sputum yielded the highest pathogen detection rate for both CAIs and HAIs, while oral specimens showed the lowest. Pulmonary fungal infections predominated, whereas bacterial infections were more common in blood samples (**Figures 1A, B**).

**Table 3:** Pathogens identified in patients hospitalized for SFTS with co-infection.

CAIs	No. (%)	HAIs	No. (%)
Pathogen	(n=63)	Pathogen	(n=39)
*Aspergillus* genus	13 (20.6)	*A. fumigatus*	6 (15.4)
*A. fumigatus*	10 (15.9)	*Aspergillus* genus	3 (7.7)
*S.hominis*	4 (6.3)	*A. baumanii*	3 (7.7)
*K.pneumoniae*		*S.aureus*	
CRKP	3 (4.8)	MSSA	1 (2.6)
ESBLs	1 (1.6)	MRSA	1 (2.6)
*S. epidermidis*	4 (6.3)	*A.flavus*	2 (5.2)
*E.faecium*	3 (4.8)	*Mucorales*	2 (5.2)
*A. baumanii*	2 (3.2)	*K.pneumoniae*	
*A.flavus*	2 (3.2)	CRKP	2 (5.2)
*Mycoplasma*	2 (3.2)	*Mycoplasma*	2 (5.2)
*P.aeruginosa*	2 (3.2)	*P.aeruginosa*	2 (5.2)
*A.nidulans*	1 (1.6)	*E.faecium*	2 (5.2)
*A.terreus*	1 (1.6)	*Candida*	2 (5.2)
*P.maltophila*	1 (1.6)	*Rhizopus*	1 (2.6)
*B.cereus*	1 (1.6)	*P.maltophila*	1 (2.6)
*S.aureus*		*E.aerogen*	1 (2.6)
MRSA	1 (1.6)	*Enterobacter cloacae* complex	
*Burkholderia cenocepacia*	1 (1.6)	ESBLs	1 (2.6)
*Burkholderia pluvialis*	1 (1.6)	*S.haemolyticus*	1 (2.6)
*E.cloacae*		*Rickettsial tsutsugamushioi*	1 (2.6)
ESBLs	1 (1.6)	*Actinomyces neuii*	1 (2.6)
*Oral Streptococci*	1 (1.6)	*A.pittii*	1 (2.6)
*S.warneri*	1 (1.6)	*E.cloacae*	
*Burkholderia pluvialis*	1 (1.6)	ESBLs	1 (2.6)
*Corynebacterium striatum*	1 (1.6)	*P. putida*	1 (2.6)
*C.glabrata*	1 (1.6)	*Influenza virus*	1 (2.6)
*S.anginosus*	1 (1.6)		
*Candida*	1 (1.6)		
*Influenza virus*	1 (1.6)		
HPIVS	1 (1.6)		

CAIs, Community-acquired infections; HAIs, Hospital-associated infections; CRKP, Carbapenem-Resistant *Klebsiella pneumoniae*; ESBLs, extended-spectrumβ-lactamases; MRSA, methicillin-resistant *staphylococcus aureus*; MSSA, methicillin sensitive *staphylococcus aureus*; HPIVS, human parainfluenza virus.

Bacteria were the most frequently identified pathogens in both CAIs and HAIs (**Table 3**), accounting for 32 types (50.8%) and 22 types (56.4%), respectively. Fungi followed, with 29 types (46.0%) in CAIs and 16 types (41.0%) in HAIs (**Figure 1B**). Only two viral pathogens were detected in CAIs: influenza A virus and human parainfluenza virus (HPIVS). In contrast, HAIs yielded only influenza A virus. The *Aspergillus genus* was identified in 28 cases (59.6%, 28/47) of CAIs and 18 cases (52.9%, 18/34) of HAIs. In pulmonary infections, *Aspergillus genus* frequently presented as either a single infection or a bacterial co-infection, whereas sole bacterial infections were less common (**Figures 1C, D**) (**Supplementary Table S1**).

In CAIs, we detected 1 strain of methicillin-resistant *s. aureus* (MRSA), 1 strains of extended-spectrumβ-lactamases (ESBLs)-producing *E.cloacae*, 1 strains of extended-spectrumβ-lactamases (ESBLs)-producing *K. pneumoniae*, and 3 strains of carbapenem-resistant *K. pneumoniae* (CRKP). While in HAIs, we detected 2 strains of MRSA and 1 strain of methicillin sensitive *s. aureus* (MSSA), 2 strains of ESBLs-producing *Enterobacteriaceae*, and 2 strains of CRKP (**Table 3**).

### 3.4 Risk factor analysis in SFTS patients with CAIs

Given the poorer clinical manifestations and outcomes observed in SFTS patients with CAIs, we conducted multivariate logistic regression analyses to identify risk factors associated with CAIs in SFTS patients. Prior to conducting multivariate logistic regression analysis, we first performed Lasso regression analysis on all variables with significant variables identified in **Table 1** and **Table 4**. This preprocessing step aimed to achieve the dual objectives of variable selection and model regularization. Simultaneously eliminate multicollinearity among variables and prevent mutual interference during subsequent logistic regression analysis, thereby selecting variables with genuine predictive value (**Figure 2**). The Lasso regression analysis ultimately retained 10 variables with non-zero coefficients: diarrhea, cerebral infarction, ribavirin, antibiotic therapy, antifungal therapy, Transfer to ICU, myocardial injury, amylase, D-Dimer, and A/G ratio <1.05 (**Supplementary Table S2**).

**Table 4:** Hematological parameters of SFTS patients.

Variables	Total cohort (N=231)	Non-co-infection (n=150)	CAIs (n=47)	HAIs (n=34)	*P* ^a^ Value	*P ^b^* Value
Hematological parameters						
LDH (U/L)	578.00 (812.93-1118.47)	552.50 (750.70-1096.19)	669.00 (780.37-1787.99)	539.00 (542.03-894.68)	0.066	0.865
CK(U/L)	332.00 (603.51-1487.06)	326.00 (522.60-956.24)	346.00 (-30.02-2279.67)	346.00 (-170.13-4750.13)	0.696	0.355
CK-MB(ng/mL)	15.00 (20.24-31.31)	13.50 (18.32-28.38)	17.00 (11.49-43.77)	19.00 (11.64-56.34)	0.315	0.644
cTnI(ng/L)	0.04 (0.04-0.85)	0.04 (0.06-0.55)	0.06 (0.09-0.41)	0.03 (-1.24-3.93)	0.007	0.976
Mb(μg/L)	101.00 (181.69-244.89)	97.25 (166.90-240.74)	91.00 (126.17-259.81)	153.60 (172.73-393.05)	0.818	0.158
WBC(x10^9^)	2.68 (3.39-4.32)	2.62 (3.00-3.99)	3.36 (3.71-6.25)	2.34 (2.40-5.41)	0.121	0.439
Neut(x10^9^)	1.75 (2.34-3.13)	1.68 (2.00-2.87)	2.03 (2.53-4.64)	1.61 (1.60-4.21)	0.132	0.699
Lym(x10^9^)	0.66 (0.75-0.93)	0.69 (0.72-0.90)	0.65 (0.74-1.33)	0.53 (0.46-0.93)	0.513	0.089
Mono(x10^9^)	0.15 (0.21-0.31)	0.15 (0.19-0.26)	0.15 (0.21-0.48)	0.14 (0.05-0.54)	0.529	0.462
Eos(x10^9^)	0.00 (0.00-0.01)	0.00 (0.00-0.01)	0.00 (0.00-0.01)	0.00 (0.00-0.01)	0.630	0.587
Bas(x10^9^)	0.01 (0.01-0.02)	0.01 (0.01-0.02)	0.01 (0.01-0.02)	0.00 (0.00-0.01)	0.740	0.074
RBC(x10^12^)	4.33 (4.14-4.83)	4.31 (4.03-4.09)	4.46 (4.12-4.52)	4.36 (4.15-4.58)	0.829	0.635
Hb(g/L)	130.39 ± 1.24	129.98 ± 1.46	130.36 ± 3.20	132.27 ± 3.19	0.960	0.531
PLT(x10^9^)	59.50 (60.47-70.23)	60.50 (60.21-72.90)	55.00 (51.35-64.74)	55.00 (54.26-85.74)	0.590	0.531
Hct(%)	38.68 ± 0.34	38.52 ± 0.40	38.79 ± 0.90	39.26 ± 0.85	0.903	0.502
CRP(mg/L)	4.71 (7.81-13.13)	4.30 (6.20-12.67)	4.32 (6.96-22.92)	6.22 (5.95-11.96)	0.257	0.231
PCT(ng/mL)	0.16 (0.28-0.62)	0.13 (0.25-0.42)	0.20 (0.09-1.16)	0.17 (-0.16-1.63)	0.061	0.339
ALT(U/L)	56.50 (76.43-100.51)	53.50 (72.36-101.95)	60.00 (65.57-128.21)	63.00 (55.10-110.51)	0.468	0.454
AST(U/L)	132.00 (206.12-295.37)	124.00 (186.32-286.03)	165.00 (178.26-467.67)	157.00 (136.60-296.88)	0.159	0.605
ALP(U/L)	74.00 (80.77-99.85)	73.00 (76.59-101.85)	85.00 (79.16-125.31)	72.00 (66.95-90.83)	0.157	0.814
GGT(U/L)	31.00 (48.05-69.22)	28.00 (44.80-73.21)	53.00 (45.15-81.86)	34.00 (23.73-77.01)	0.051	0.648
ALB(g/L)	35.94 ± 0.41	36.37 ± 0.47	33.63 ± 1.06	37.18 ± 1.04	0.016	0.336
A/G	1.27 ± 0.02	1.29 ± 0.22	1.11 ± 0.05	1.34 ± 0.05	0.002	0.417
HCO3^-^(mmol/L)	22.40 (21.56-22.67)	22.40 (21.71-22.97)	23.10 (20.62-23.48)	21.20 (19.43-22.92)	0.500	0.142
BUN(mmol/L)	6.36 (6.80-7.98)	6.13 (6.41-7.80)	6.46 (6.47-9.03)	6.23 (6.18-10.16)	0.195	0.151
CRE(μmol/L)	74.55 (80.27-97.90)	73.75 (77.24-94.10)	78.50 (69.11-111.12)	78.40 (63.57-141.97)	0.724	0.446
eGFR(mL/(min·1.73m^2^))	86.00 (77.66-84.29)	89.00 (78.76-86.92)	85.00 (69.57-84.79)	84.00 (68.73-87.03)	0.223	0.466
UA(μmol/L)	264.55 (278.94-314.37)	261.30 (267.33-310.46)	295.00 (268.53-350.10)	317.00 (262.05-365.36)	0.348	0.282
AMY(U/L)	93.50 (105.30-126.87)	91.50 (97.49-123.78)	102.00 (107.20-167.49)	95.00 (92.26-130.12)	0.028	0.394
LPS(U/L)	414.00 (483.34-610.01)	406.00 (439.46-600.91)	600.00 (480.36-720.00)	334.00 (402.95-778.85)	0.026	0.751
FBG(mmol/L)	6.89 (7.69-8.74)	6.85 (7.51-8.73)	7.36 (7.29-9.54)	6.70 (6.47-10.24)	0.375	0.872
K^+^(mmol/L)	3.83 (3.77-3.91)	3.81 (3.76-3.93)	3.79 (3.60-3.92)	3.87 (3.70-4.14)	0.380	0.860
Na^+^(mmol/L)	133.35 (133.15-134.39)	133.60 (133.06-134.52)	133.40 (132.58-136.03)	132.70 (131.42-134.49)	0.927	0.188
Cl^-^(mmol/L)	99.00 (98.95-100.31)	99.35 (98.72-100.24)	98.60 (98.44-102.34)	98.80 (97.39-101.14)	0.988	0.534
APTT(s)	47.65 (48.76-56.44)	46.65 (47.25-52.35)	50.00 (48.19-59.82)	48.20 (40.19-85.99)	0.203	0.067
D-D(μg/mL)	2.49 (3.79-5.05)	2.15 (3.23-4.73)	3.41 (4.15-7.61)	2.99 (3.08-5.74)	0.006	0.173
FDP(μg/mL)	8.51 (14.73-22.04)	7.38 (12.46-21.41)	11.26 (14.58-35.41)	9.28 (10.30-21.28)	0.031	0.217
TT(s)	21.45 (25.72-32.77)	21.05 (24.63-34.46)	21.80 (24.42-37.78)	21.50 (20.19-30.60)	0.246	0.864
FIB(g/L)	2.65 (2.66-2.85)	2.64 (2.62-2.84)	2.71 (2.57-3.18)	2.71 (2.48-2.91)	0.673	0.800

CAIs, Community-acquired infections; HAIs, hospital-acquired infections; LDH, Lactate dehydrogenase; CK, Creatine phosphokinase; CK-MB, Creatine kinase isoenzyme; cTnI, Cardiac troponin I; Mb, Myoglobin; WBC, White blood cell; Neut, Neutrophils; Lym, Lymphocytes; Mono, Monocytes; Eos, Eosinophils; Bas, Basophils; RBC, Red blood cell; Hb, Hemoglobin; PLT, Platelet; Hct, Hematocrit; CRP, C-reactive protein; PCT, Procalcitonin; ALT, Alanine aminotransaminase; AST, Aspartate aminotransferase; ALP, Alkaline phosphatase; GGT, Gamma‐glutamyl transferase; ALB, Albumin; A/G, Albumin/globulin ratio; BUN, Blood urea nitrogen; CRE, Creatinine; eGFR, estimated glomerular filtration rate; UA, Uric acid; AMY, Amylase; LPS, Lipase; FBG, Fasting blood glucose; APTT, Activated partial thromboplastin time; D-D,D‐dimer; FDP, fibrinogen degradation product; TT, Thrombin time; FIB, Fibrinogen; HCO3^-^, hydrogen carbonate ion.

^a^ Comparison between non-co-infection and CAIs; ^b^ Comparison between non-co-infection and HAIs. Statistical analysis was performed using Student’s t-test or Mann-Whitney U test based on the normal distribution characteristics of data.

**Figure 2:**
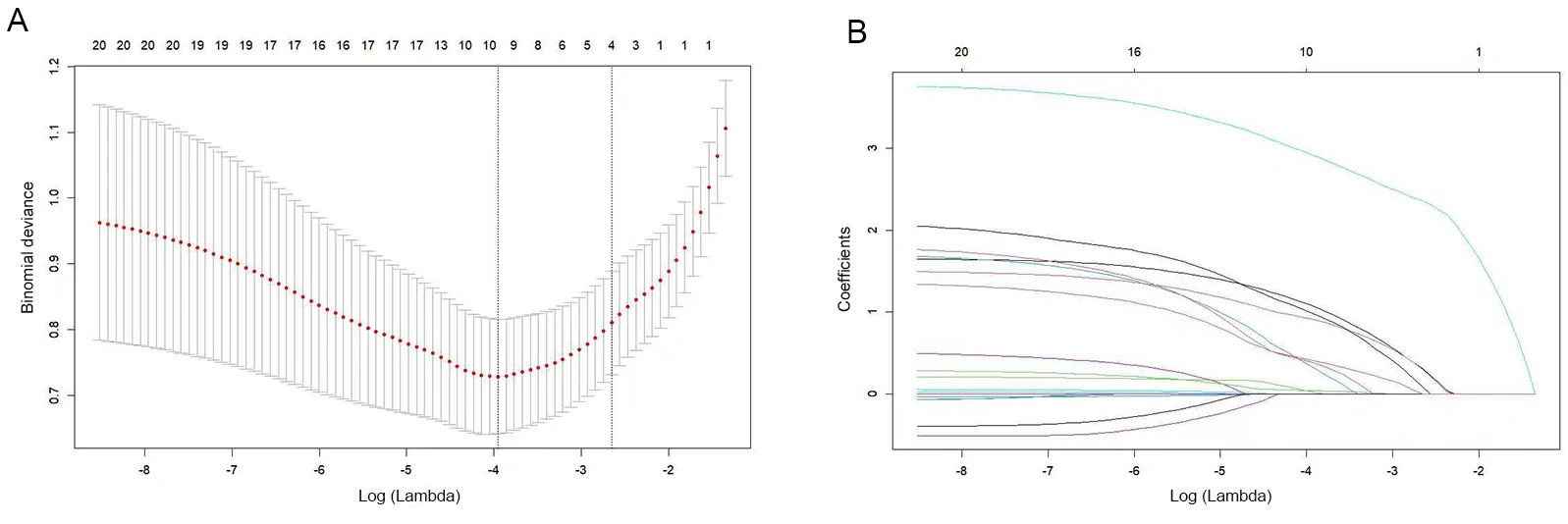
Lasso logistic regression analyses of risk factors for CAIs in SFTS patients. **(A)** Binomial deviance measures the goodness-of-fit of a binomial distribution model. The vertical line on the left represents the lambda value corresponding to the optimal fit of the binomial distribution, the vertical line on the right indicates the lambda value within one standard error of the optimal value; **(B)** Variable Screening trajectories of Lasso regression analysis. In Lasso regression analysis, non-zero coefficients indicate that the selected variables have predictive value, whereas zero coefficients represent that the selected variables lack significance.

Multivariate logistic regression analysis using variables selected by Lasso regression demonstrated that diarrhea (OR 4.826 [1.695-13.737]), history of cerebral infarction (OR 4.347 [1.482-12.749]), antifungal therapy (OR 46.076 [9.171-231.495]), transfer to ICU (OR 9.551 [1.338-68.183]), D-Dimer (OR 1.110 [1.010-1.220]), A/G ratio <1.05 (OR 5.116 [1.011-25.889]) were independent risk factors for the occurrence of CAIs in SFTS patients (**Figure 3**).

**Figure 3:**
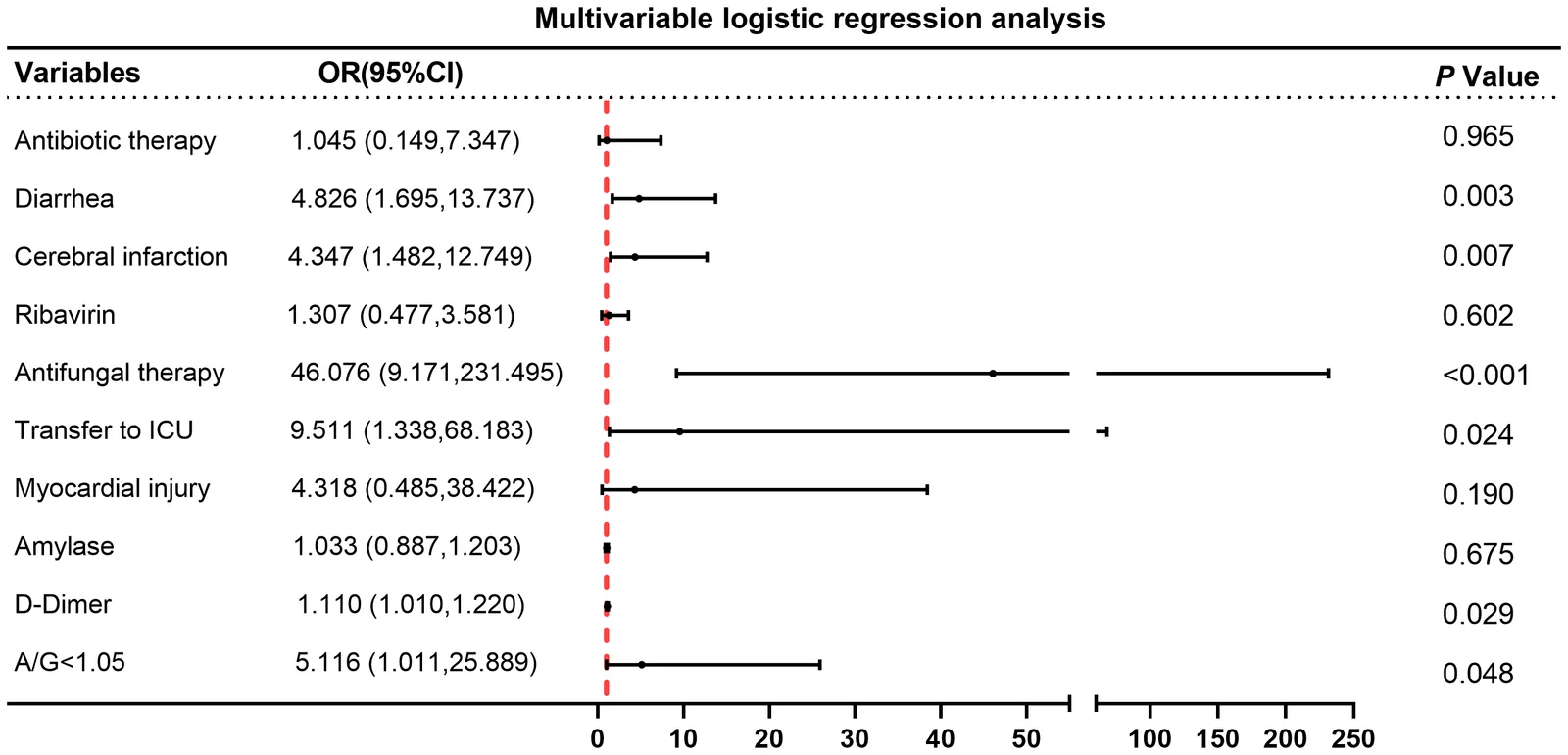
Multivariable logistic regression analyses of risk factors for CAIs in SFTS patients.

Furthermore, ROC curve analysis revealed that an A/G ratio <1.05 had supportive risk-assessment value for CAIs in SFTS patients (AUC 0.714, 95%CI: 0.626-0.832, **Figure 4**), indicating that the A/G ratio could be considered as a supportive predictive indicator for SFTS patients with CAIs. the discriminative ability of A/G ratio <1.05 for CAIs-related pulmonary fungal infections was the strongest, with an average area under the ROC curve (AUC) of 0.799 (95% CI: 0.702-0.896), and the average area under the ROC curve (AUC) for predicting CAIs-related non-bloodstream infections was 0.729 (95%CI: 0.626-0.832). However, the predictive performance of A/G ratio <1.05 for CAIs-related bloodstream infections (AUC 0.67, 95%CI: 0.493-0.847) and no pulmonary fungal infections (AUC 0.567, 95%CI: 0.415-0.719) was relatively weak. The predictive ability of D-Dimer level for SFTS with CAIs was relatively weak (AUC 0.647, 95%CI: 0.560-0.735) (**Supplementary Figure S1**).

**Figure 4:**
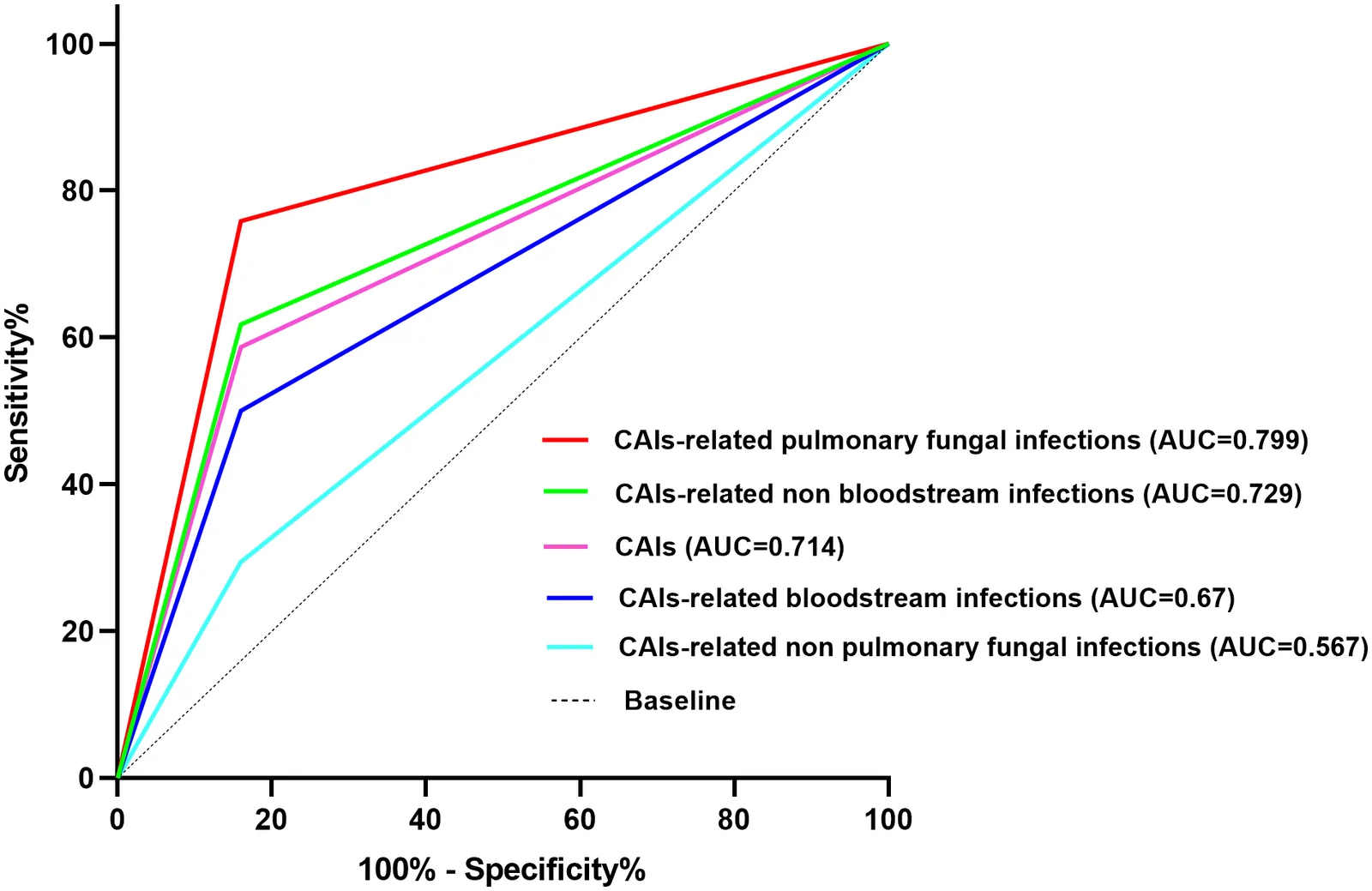
Predictive performance of A/G ratio <1.05 for CAIs in SFTS patients.

### 3.5 Survival analysis of SFTS patients with co-infection

Kaplan-Meier survival analysis indicated that the survival time of SFTS patients with CAIs was significantly shorter than those with HAIs, suggesting a poor prognosis for SFTS patients with CAIs. We further analyzed the impact of antifungal therapy on severe/critically ill patients with CAIs-related fungal infections and HAIs-related fungal infections. Among 27 severe/critically ill SFTS patients with CAIs-related fungal infections, 20 received antifungal therapy, which significantly extended the survival time of patients with CAIs-related fungal infections (*p* = 0.008). In contrast, antifungal therapy did not have a significant effect on improving the survival time of severe/critically ill patients with HAIs-related fungal infections (*p* = 0.453). Compared to SFTS patients without co-infection, SFTS patients with HAIs-related pulmonary infections had a longer survival time (*p* = 0.039), however, the survival time of SFTS patients with CAIs-related pulmonary infections was not significantly different from those without co-infection (*p* = 0.812), possibly due to receiving aggressive antibiotic and antifungal therapy (**Figure 5**).

**Figure 5:**
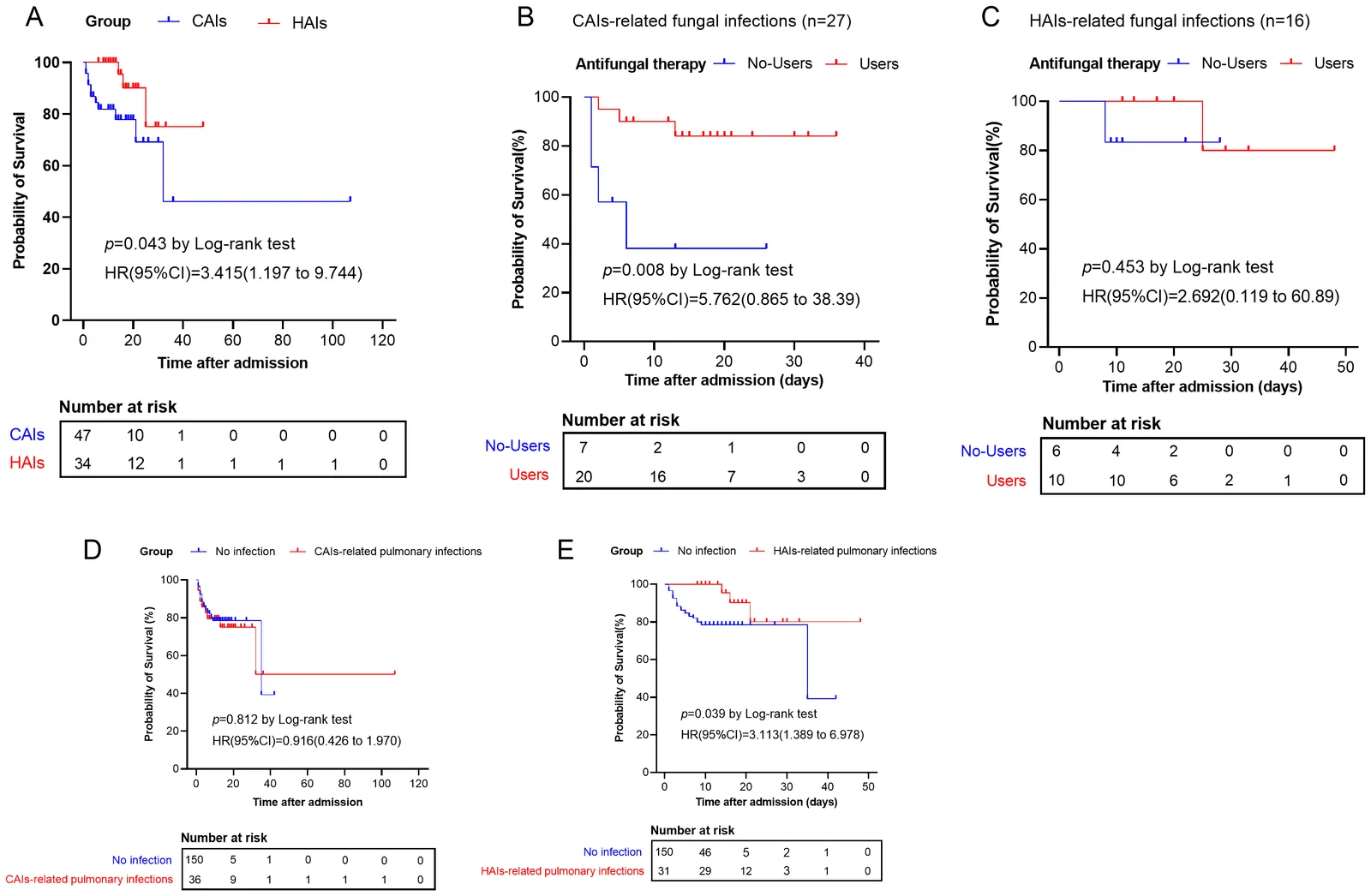
Survival outcomes in SFTS patients with different co-infection types. **(A)** Kaplan-Meier survival curves comparing CAIs and HAIs. **(B)** Impact of antifungal therapy on survival in SFTS patients with CAIs-related fungal infections. **(C)** Effect of antifungal therapy on survival in SFTS patients with HAIs-related fungal infections. **(D)** Survival comparison between SFTS patients with CAIs-related pulmonary infections and those without co-infection. **(E)** Survival comparison between SFTS patients with HAIs-related pulmonary infections and those without co-infection.

### 3.6 Correlation between A/G ratio and hematological parameters in SFTS patients with CAIs

Correlation analysis results showed that the A/G ratio was significantly positively correlated with PLT (r=0.372, *p* < 0.001), ALB (r=0.707, *p* < 0.001), HCO3- (r=0.173, *p* = 0.015) and significantly negatively correlated with LDH (r=-0.421, *p* < 0.001), CK (r=-0.205, *p* = 0.004), CK-MB (r=-0.141, *p* = 0.048), cTnI (r=-0.375, *p* < 0.001), Mb (r=-0.205, *p* = 0.004), WBC (r=-0.331, *p* < 0.001), Neut (r=-0.277, *p* < 0.001), Lym (r=-0.274, *p* < 0.001), Mono (r=-0.272, *p* < 0.001), Bas (r=-0.324, *p* < 0.001), CRP (r=-0.213, *p* = 0.003), PCT (r=-0.275, *p* < 0.001), ALT (r=-0.356, *p* < 0.001), AST (r=-0.395, *p* < 0.001), ALP (r=-0.276, *p* < 0.001), GGT (r=-0.41, *p* < 0.001), AMY (r=-0.286, *p* < 0.001), LPS (r=-0.325, *p* < 0.001), FBG (r=-0.307, *p* < 0.001), Cl- (r=-0.226, *p* = 0.001), APTT (r=-0.252, *p* < 0.001), D-D (r=-0.275, *p* < 0.001), FDP (r=-0.297, *p* < 0.001) and TT (r=-0.357, *p* < 0.001).However, the A/G ratio is not significantly correlated with RBC, Hb, Hct, Eos, eGFR, CRE, UA, BUN, K+, Na+, FIB (*p*>0.05) (**Figure 6**).

**Figure 6:**
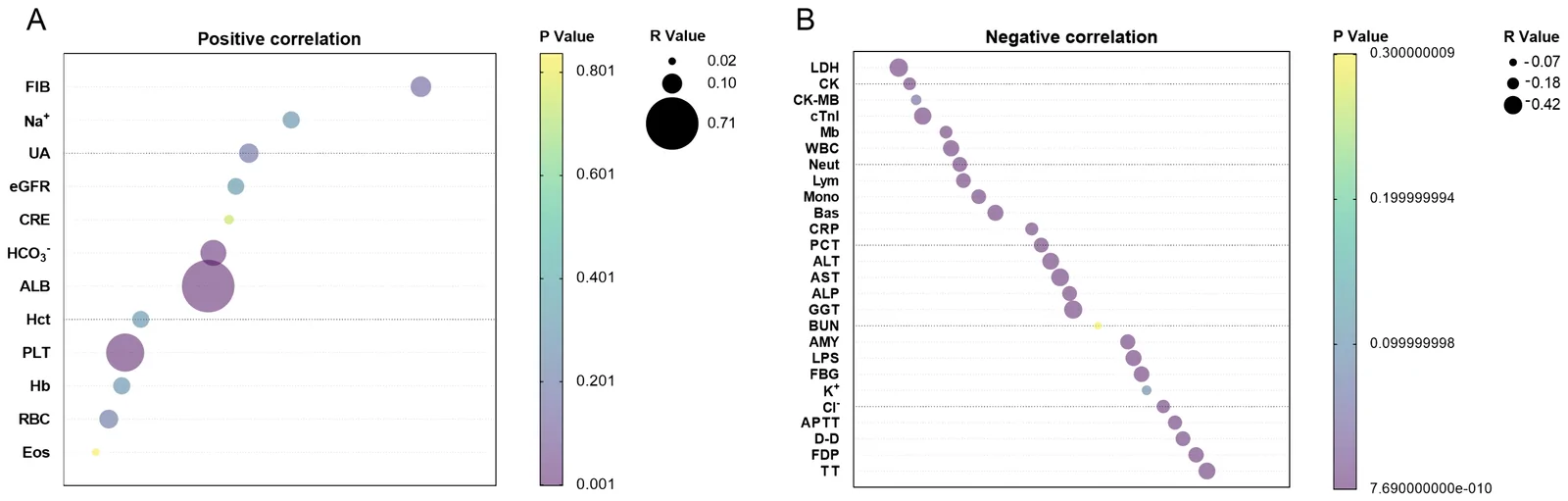
Correlation between A/G ratio and laboratory parameters in hospitalized SFTS patients. **(A)** Blood parameters showing positive correlation with A/G ratio. **(B)** Blood parameters showing negative correlation with A/G ratio.

## 4 Discussion

The high morbidity and mortality rates associated with SFTS present a significant public health threat. When SFTS patients develop co-infection, particularly those with severe SFTS clinical management becomes more challenging, potentially exacerbating disease progression, delaying recovery, and increasing mortality risk (Kubota et al., 2024). Early identification of risk factors for co-infection in SFTS patients is crucial for timely intervention and improved outcomes.

Although SFTS is a tick-borne illness, many patients lack a documented history of tick exposure or bites. Consequently, they often seek medical care only after symptoms such as fever, vomiting, and hemorrhage. By this stage, the immune system is frequently compromised (Yang et al., 2022), and comorbidities or advanced age further predispose patients to secondary infections. Community-acquired pathogens (respiratory, gastrointestinal, or cutaneous) and hospital-acquired exposures (e.g., antibiotic-induced microbiome disruption) (Vaughn et al., 2021) also contribute to infection risk. Despite these challenges, evidence regarding SFTS-associated co-infections remains limited, particularly concerning the clinical differences between CAIs and HAIs. In our cohort, 35.1% of patients with SFTS developed co-infection, with CAIs occurring more frequently than HAIs (20.3% vs 14.7%). Compared with patients without co-infection, those with CAIs had more severe clinical manifestations, a higher frequency of multiple-organ dysfunction, and higher mortality (23.4%). These findings are consistent with previous retrospective studies reporting a substantial burden of CAIs in SFTS (Ge et al., 2022). However, the higher mortality observed among patients with CAIs in our cohort contrasts with previous reports of poorer outcomes among patients with HAIs. This discrepancy may reflect differences in patient characteristics, disease severity, infection profiles, or treatment strategies across studies (Xiong et al., 2022; Chen et al., 2024). Further prospective studies are warranted to clarify the factors underlying the different clinical trajectories and outcomes associated with CAIs and HAIs in patients with SFTS.

Existing studies report varying data on pathogens coinfecting with SFTS. Viral co-infection with SFTS are rare, while bacterial co-infection—though more common—are typically less severe than fungal co-infection, particularly Aspergillus, which significantly impacts patient outcomes (Song et al., 2023; Zhao et al., 2025). The predominant *Aspergillus* species identified in SFTS co-infection include *A. fumigatus*, *A. flavus*, *A. terreus*, with *A. fumigatus* and *A. flavus* being the most prevalent—a finding consistent with previous research. Aspergillus co-infection poses a critical threat to SFTS patients, often manifesting as invasive pulmonary aspergillosis (IPA), which accelerates disease progression and increases mortality (Iwao et al., 2021). Our research suggested that early antifungal therapy reduced mortality in CAIs patients, but showed no significant effect in HAIs cases. Notably, HAIs patients with IPA had markedly shorter survival times than non-coinfected SFTS patients, underscoring the need for prompt, aggressive antifungal treatment in CAIs cases.

Given the poorer outcome in SFTS patients with CAIs, we identified several predictive risk factors. Diarrhea at admission—a known prognostic marker for SFTS (He et al., 2020)—was more frequent in CAIs patients. SFTSV-induced systemic inflammation compromise intestinal mucosal barrier function, facilitating opportunistic pathogen translocation (Liu et al., 2025). Clinically, diarrhea may also obscure infection symptoms, delaying diagnosis. While cerebral infarction history is not yet established as SFTS risk factor, our data revealed it as an independent predictor of CAIs. Post-infarction chronic inflammation may impair antiviral immunity (Xu et al., 2021), and antiplatelet therapies (e.g., aspirin) could exacerbate SFTS-associated thrombocytopenia. Further studies are required to determine whether previous cerebral infarction has clinical utility for risk stratification. The co-infection of fungus elevates the mortality rate of SFTS (Li et al., 2018). Multivariate analysis confirmed that CAIs patients were more frequently to receive antifungals, which improved survival aligning with Hu et al.’s findings (Hu et al., 2021). ICU-admitted SFTS patients, especially those with severe disease requiring invasive procedures (e.g., mechanical ventilation, central venous catheterization) (Bertoni et al., 2020; Cosme et al., 2024), are a higher risk for co-infection (Wang G. et al., 2024; Sun et al., 2025). Our data are consistent with this, with CAIs patients more likely to require ICU care, emphasizing the need for early infection screening and immunomodulatory therapy. Elevated D-dimer levels—linked to hypercoagulability and poor prognosis (Terpos et al., 2020; Xia et al., 2024) emerged as an independent CAIs predictor, warranting close monitoring during hospitalization. Our study found that the elevation of blood D-dimer on admission in SFTS patients was an independent risk factor for predicting CAIs in SFTS patients. Therefore, changes in these clinical indicators should be closely monitored during the hospitalization of SFTS patients to achieve early identification and treatment of SFTS patients with CAIs.

The A/G ratio is commonly used to evaluate inflammation, infection, liver injury, nutritional index, tumor prognosis, etc (Suh et al., 2014; Cattaneo et al., 2021; Zhang et al., 2022; Dong et al., 2024; Hu et al., 2025). In our analysis of risk factors for SFTS patients with CAIs, we found that an A/G ratio <1.05 at admission is an important risk factor for predicting the occurrence of CAIs in SFTS patients. According to the currently reported infection data of SFTS patients, our study is the first to assess the potential prognostic value of the A/G ratio in SFTS. The results of multivariate logistic regression analysis indicated that an A/G ratio <1.05 was independently associated with CAIs and showed moderate discriminatory performance. On one hand, for SFTS patients, liver function impairment is often manifested during acute infection or inflammatory states. Impaired hepatic synthetic function leads to a decrease in albumin, while the liver may increase the production of acute phase reactants, such as globulin and C-reactive protein, thereby reducing the A/G ratio. If complicated with other pathogen infections, such as fungi or bacteria, it might exacerbate the systemic inflammatory response, leading to a more pronounced increase in globulin and a decrease in albumin, making the A/G ratio even lower. On the other hand, the unique value of the A/G ratio lies precisely in its core focus on the dynamic albumin-to-globulin ratio, with its underlying mechanism involves the fact that during disease progression, albumin depletion and abnormal globulin elevation typically precede significant organ dysfunction and substantial fluctuations in inflammatory markers. The A/G ratio may demonstrate high sensitivity in capturing these early imbalance signals, thereby filling a critical gap in established indicator systems for monitoring metabolic-immune homeostasis. Therefore, the A/G ratio may serve as an early marker indicating the risk of CAIs in SFTS patients. While the receiver operating characteristic (ROC) curves for the A/G ratio demonstrated its association with co-infection, the area under the curve (AUC) values of 0.71~0.80 indicated that it had limited utility as a standalone predictive tool. However, we would like to emphasize that the primary purpose of highlighting the A/G ratio in our study was not necessarily to propose it as a sole screening instrument. Even with an AUC in the moderate range, such a biomarker can still be valuable for risk stratification (Chen et al., 2025). A/G ratio should be considered as a supportive predictor rather than a decisive predictor in clinical work. A/G ratio has significant implications for the treatment and prevention and control of SFTS with co-infection, especially in areas where SFTS is prevalent. Through the A/G ratio at admission, high-risk groups for co-infection can be identified early and quickly. Therefore, future studies should evaluate whether incorporation of the A/G ratio into clinical decision-making algorithms improves patient management. Compared to culture and mNGS, it is a readily available and inexpensive supplementary marker (Tan et al., 2025). Moreover, the A/G ratio, as a potential biomarker for early screening of SFTS co-infection, has significant importance in areas with limited medical resources.

In addition, we also found a significant correlation between the A/G ratio and various clinical blood parameters. Among them, the A/G ratio is positively correlated with platelets. During the acute infection of SFTSV, platelet reactivity increases to promote hemostasis, and the A/G ratio, as an indirect indicator of the acute phase response, may change synchronously with this process. The positive correlation between the A/G ratio and HCO3^-^ suggests that the A/G ratio may be involved in the regulation of acid-base balance. The A/G ratio is significantly negatively correlated with inflammation markers such as CRP, PCT, Neut (Yuwen et al., 2017; Wu et al., 2019), tissue damage markers such as LDH, CK, cTnI (Mansour et al., 2023), coagulation dysfunction indicators such as D-Dimer, FDP, TT, and liver and kidney dysfunction indicators such as ALT, AST, ALP, GGT, as well as AMY (a marker of pancreatic injury). This indicates that the A/G ratio may serve as a mirror indicator for the systemic inflammatory response and multiple organ dysfunction in SFTS patients (Chu et al., 2024). The A/G ratio has no significant correlation with red blood cell parameters such as RBC, Hb, Hct, and eGFR, electrolytes (K^+^, Na^+^), indicating that its changes are independent of anemia or basic renal function. These results suggest that the abnormality of the A/G ratio is more dependent on the liver’s synthetic function and systemic inflammatory response, rather than simple blood dilution or concentration effects. In summary, the A/G ratio, as an economical and easy-to-measure laboratory indicator, should be closely monitored in clinical work to provide an important basis for the timely treatment and prognosis assessment of SFTS patients.

This study has some limitations, due to the retrospective analysis, the sample size is limited, and the disease severity of SFTS patients, host factors (such as underlying diseases, age, immune status), invasive operations, length of hospital stay, etc., may all affect the antifungal treatment effect of SFTS patients with co-infection. Therefore, when evaluating the potential biomarkers of SFTS co-infection and the therapeutic effect against co-infection, various interference factors need to be comprehensively considered, and the results may be biased. Secondly, co-infection caused by diagnostic and therapeutic procedures, environmental exposure, or activation of colonized bacteria, although occurring within 48 hours of admission, may actually be hospital related infections. Therefore, in future research, all information should be comprehensively considered to make judgments. Thirdly, we did not dynamically analyze the dynamic changes of clinical parameters in SFTS patients. Fourthly, the conclusion that the A/G ratio was a potential biomarker is speculative and has not been further validated for its mechanism. Finally, although this study established the predictive value of the A/G ratio in the initial cohort, the generalizability of these findings is limited by the single-center design characterized by homogeneous population features, standardized diagnostic and therapeutic protocols, and sample homogeneity, which collectively fail to fully replicate the complexity of clinical scenarios. Moreover, as a retrospective observational study, although a low A/G ratio was associated with CAIs, the present study does not demonstrate that its incorporation into clinical decision-making improves patient outcomes. Therefore, our research results cannot fully represent the clinical characteristics of SFTS patients, and it is necessary to expand the sample size and extend the time span of data analysis in the future.

## 5 Conclusion

Our study examined the clinical characteristics of SFTS patients, focusing on differences between those with or without co-infection. The results demonstrated that SFTS patients with CAIs had more severe clinical manifestations and worse outcomes compared to those without co-infection or with HAIs. While antifungal treatment significantly prolonged survival in SFTS patients with CAIs-related fungal infections, it had no effect on those with HAIs-related fungal infections. Univariate and multivariate logistic regression analyses identified several independent risk factors for CAIs in SFTS patients, including diarrhea on admission, a history of cerebral infarction, antifungal therapy, ICU admission, and elevated D-Dimer levels. Additionally, an A/G ratio <1.05 was associated with CAIs and may have potential value as a supportive risk-assessment marker. Given these findings, clinicians should closely monitor these indicators to better assess disease progression and treatment efficacy in SFTS patients.

## Data Availability

The original contributions presented in the study are included in the article/**Supplementary Material**. Further inquiries can be directed to the corresponding author.

## Supplementary material

Supplementary Figure 1 Predictive performance of D-Dimer for CAIs in SFTS patients.
