# Supplementary material for: The pathogenic and clinical characteristics of community-acquired and hospital-acquired co-infection in patients hospitalized with severe fever with thrombocytopenia syndrome
Source: Front Cell Infect Microbiol. 2026 Oct 29;16:1940837. doi: 10.3389/fcimb.2026.1940837 (PMC13754672; doi:10.3389/fcimb.2026.1940837)
Supplement: Supplementary file 1 [file DataSheet1.docx]

1. Statistical analysis data of the ROC curve for SFTS patients with CAIs.

| **Area under the ROC curve** | **CAIs** | **Pulmonary fungal coinfections** | **Non bloodstream coinfections** | **Bloodstream coinfections** | **Non pulmonary fungal coinfections** | **D-Dimer** |
| --- | --- | --- | --- | --- | --- | --- |
| **Area** | 0.7135 | 0.7993 | 0.7288 | 0.67 | 0.5671 | 0.6472 |
| **Std. Error** | 0.04708 | 0.04938 | 0.05268 | 0.09028 | 0.07764 | 0.04456 |
| **95% confidence interval** | 0.6212 to 0.8058 | 0.7025 to 0.8961 | 0.6256 to 0.8321 | 0.4930 to 0.8470 | 0.4149 to 0.7192 | 0.5598 to 0.7345 |
| **P value** | <0.0001 | <0.0001 | <0.0001 | 0.0504 | 0.3655 | 0.0024 |

Survival Curves analysis data of SFTS patients with co-infection.

| **Comparison of Survival Curves** |  |  |  |  |  |
| --- | --- | --- | --- | --- | --- |
| **Log-rank (Mantel-Cox) test** | **CAIs vs HAIs** | **CAIs-related fungal infections** | **HAIs-related fungal infections** | **CAIs-related pulmonary infections vs No infection** | **HAIs-related pulmonary infections vs No infection** |
| Chi square | 4.102 | 7.066 | 0.5628 | 0.05656 | 4.265 |
| df | 1 | 1 | 1 | 1 | 1 |
| P value | 0.0428 | 0.0079 | 0.4531 | 0.812 | 0.0389 |
| **Hazard Ratio (log rank)** |  |  |  |  |  |
| Ratio (and its reciprocal) | 3.415 | 5.762 | 2.692 | 0.9161 | 3.113 |
| 95% CI of ratio | 1.197 to 9.744 | 0.8650 to 38.39 | 0.1190 to 60.89 | 0.4260 to 1.970 | 1.389 to 6.978 |

2. Statistical data on the correlation analysis between A/G ratio and hematological parameters in SFTS patients with CAIs.

| **Hematological parameters** | **Correlation coefficient R** | **P Value** |
| --- | --- | --- |
| EOS | 0.015 | 0.837316 |
| RBC | 0.092 | 0.198903 |
| HB | 0.076 | 0.290135 |
| PLT | 0.372 | 7.96E-08 |
| Hct | 0.074 | 0.299448 |
| ALB | 0.707 | 4.57E-31 |
| HCO3- | 0.173 | 0.015379 |
| CRE | 0.024 | 0.737416 |
| eGFR | 0.072 | 0.315121 |
| UA | 0.097 | 0.175935 |
| Na | 0.076 | 0.286934 |
| FIB | 0.109 | 0.128792 |
| LDH | -0.421 | 7.69E-10 |
| CK | -0.205 | 0.003867 |
| CKMB | -0.141 | 0.04798 |
| TnI | -0.375 | 5.92E-08 |
| Mb | -0.205 | 0.003959 |
| WBC | -0.331 | 0.00000219 |
| Neut | -0.277 | 0.0000826 |
| Lym | -0.274 | 0.000101 |
| Mon | -0.272 | 0.000114 |
| Bas | -0.324 | 0.00000349 |
| CRP | -0.213 | 0.00292 |
| PCT | -0.275 | 0.000101 |
| ALT | -0.356 | 0.000000301 |
| AST | -0.395 | 9.9E-09 |
| ALP | -0.276 | 0.000088 |
| GGT | -0.41 | 2.47E-09 |
| BUN | -0.074 | 0.3005 |
| AMY | -0.286 | 0.0000496 |
| LPS | -0.325 | 0.00000344 |
| FBG | -0.307 | 0.0000117 |
| K+ | -0.121 | 0.09095 |
| Cl- | -0.226 | 0.001439 |
| APTT | -0.252 | 0.000373 |
| D-D | -0.275 | 0.0000984 |
| FDP | -0.297 | 0.0000243 |
| TT | -0.357 | 0.000000289 |

3. The efficacy of sample size
