# Supplementary material for: The pathogenic and clinical characteristics of community-acquired and hospital-acquired co-infection in patients hospitalized with severe fever with thrombocytopenia syndrome
Source: Front Cell Infect Microbiol. 2026 Oct 29;16:1940837. doi: 10.3389/fcimb.2026.1940837 (PMC13754672; doi:10.3389/fcimb.2026.1940837)
Supplement: Supplementary file 2 [file Table1.docx]

Supplementary Table S1. Pathogen information of SFTS patients with co-infection.

| **Patient ID** | **Strain name** | | | | **Strain source** | | | | | | **Pathogen positive detection method** | | | | | | | **Time of positive pathogen report after admission(hours)** | | | **Drug sensitivity** | **Antifungal therapy** | |
| --- | --- | --- | --- | --- | --- | --- | --- | --- | --- | --- | --- | --- | --- | --- | --- | --- | --- | --- | --- | --- | --- | --- | --- |
|  | **Bacteria** | **Fungus** | **Virus** | **Blood** | | **Sputum** | **BALF** | **Urine** | **Oral cavity** | **Culture** | | **mNGS** | **Gram staining** | **IgM antibody** | **Chest CT evidence** | **G test** | **GM test** | **<48** | **≥48** |  | |  |  |
| P1 |  | A. fumigatus |  |  | | yes |  |  |  | yes | |  |  |  |  |  |  | yes |  |  | | yes |  |
| P2 | K. pneumoniae |  | Influenza virus |  | |  | yes |  |  |  | | yes |  |  |  |  |  | yes |  | CRKP | |  |  |
| P3 |  | Aspergillus genus |  |  | | yes |  |  |  | yes | |  |  |  |  |  |  | yes |  |  | |  |  |
| P4 |  | Aspergillus genus |  |  | | yes |  |  |  | yes | |  |  |  |  |  |  | yes |  |  | |  |  |
| P5 | A. baumanii | A.terreus |  |  | | yes |  |  |  | yes | |  |  |  |  |  |  | yes |  |  | | yes |  |
| P6 |  | A. fumigatus |  |  | | yes |  |  |  | yes | |  |  |  |  |  |  | yes |  |  | |  |  |
| P7 | S. epidermidis | A. fumigatus |  |  | | yes |  |  |  | yes | |  |  |  |  |  |  | yes |  |  | |  |  |
| P8 |  | A. fumigatus |  |  | | yes |  |  |  | yes | |  |  |  |  |  |  | yes |  |  | | yes |  |
| P9 | S. epidermidis |  |  | yes | |  |  |  |  | yes | |  |  |  |  |  |  | yes |  |  | |  |  |
| P10 | K. pneumoniae, S. hominis |  |  | yes | |  |  | yes |  | yes | |  |  |  |  |  |  | yes |  | CRKP | |  |  |
| P11 |  | A. fumigatus |  |  | | yes |  |  |  | yes | |  |  |  |  |  |  | yes |  |  | | yes |  |
| P12 | E. faecium |  |  | yes | |  |  |  |  | yes | |  |  |  |  |  |  | yes |  |  | | yes |  |
| P13 |  | A. fumigatus |  |  | | yes |  |  |  | yes | |  |  |  |  |  |  | yes |  |  | |  |  |
| P14 | S. epidermidis |  |  | yes | |  |  |  |  | yes | |  |  |  |  |  |  | yes |  |  | |  |  |
| P15 | Mycoplasma |  |  |  | | yes |  |  |  |  | |  |  | yes |  |  |  | yes |  |  | |  |  |
| P16 | K. pneumoniae, P. maltophila, B.cereus | Aspergillus genus |  |  | | yes |  |  |  | yes | |  |  |  |  |  |  | yes |  | CRKP | | yes |  |
| P17 |  | Aspergillus genus |  |  | | yes |  |  |  | yes | |  |  |  |  |  |  | yes |  |  | | yes |  |
| P18 |  | A. fumigatus |  |  | | yes |  |  |  | yes | |  |  |  |  |  |  | yes |  |  | |  |  |
| P19 |  | Aspergillus genus |  |  | | yes |  |  |  | yes | |  |  |  |  |  |  | yes |  |  | |  |  |
| P20 |  | Aspergillus genus |  |  | | yes |  |  |  | yes | |  |  |  |  |  |  | yes |  |  | | yes |  |
| P21 |  | Aspergillus genus |  |  | | yes |  |  |  | yes | |  |  |  |  |  |  | yes |  |  | |  |  |
| P22 | E. faecium, Mycoplasma, P. aeruginosa | A. flavus |  | yes | | yes |  |  |  | yes | |  |  | yes |  |  |  | yes |  |  | | yes |  |
| P23 | S. hominis | Aspergillus genus |  | yes | | yes |  |  |  | yes | |  |  |  |  |  |  | yes |  |  | | yes |  |
| P24 |  | C. glabrata, Unknown fungus |  |  | |  |  | yes |  | yes | |  |  |  | yes |  |  | yes |  |  | |  |  |
| P25 |  | A. fumigatus |  |  | | yes |  |  |  | yes | |  |  |  |  |  |  | yes |  |  | | yes |  |
| P26 |  | A. flavus |  |  | | yes |  |  |  | yes | |  |  |  |  |  |  | yes |  |  | |  |  |
| P27 |  | Aspergillus genus |  |  | | yes |  |  |  | yes | |  |  |  |  |  |  | yes |  |  | | yes |  |
| P28 | E. faecium | A. fumigatus |  |  | | yes |  | yes |  | yes | |  |  |  |  |  |  | yes |  |  | | yes |  |
| P29 |  | A. fumigatus |  |  | | yes |  |  |  | yes | |  |  |  |  |  |  | yes |  |  | |  |  |
| P30 | S. hominis | Candida, Unknown fungus |  | yes | |  |  |  | yes | yes | |  |  |  | yes |  |  | yes |  |  | | yes |  |
| P31 | K. pneumoniae | Aspergillus genus |  |  | | yes |  |  |  | yes | |  |  |  |  |  |  | yes |  | ESBLs | | yes |  |
| P32 | A. baumanii, S. aureus | Aspergillus genus |  |  | | yes |  |  |  | yes | |  |  |  |  |  |  | yes |  | MRSA | | yes |  |
| P33 |  | Aspergillus genus |  |  | | yes |  |  |  | yes | |  |  |  |  |  |  | yes |  |  | | yes |  |
| P34 | S. anginosus |  |  |  | |  |  | yes |  | yes | |  |  |  |  |  |  | yes |  |  | |  |  |
| P35 |  | A. nidulans |  |  | | yes |  |  |  | yes | |  |  |  |  |  |  | yes |  |  | |  |  |
| P36 | Oral Streptococci |  |  | yes | |  |  |  |  | yes | |  |  |  |  |  |  | yes |  |  | | yes |  |
| P37 | P. aeruginosa, Burkholderia cenocepacia, Burkholderia pluvialis, E. cloacae, S.warneri, Corynebacterium striatum |  |  | yes | |  | yes |  |  |  | | yes |  |  |  |  |  | yes |  | ESBLs | | yes |  |
| P38 | S. hominis |  |  | yes | |  |  |  |  | yes | |  |  |  |  |  |  | yes |  |  | | yes |  |
| P39 |  | Aspergillus genus |  |  | | yes |  |  |  | yes | |  |  |  |  |  |  | yes |  |  | | yes |  |
| P40 | S. epidermidis |  |  | yes | |  |  |  |  | yes | |  |  |  |  |  |  | yes |  |  | | yes |  |
| P41 | Unknown bacteria |  |  | yes | |  |  |  |  |  | |  | yes |  |  |  |  | yes |  |  | |  |  |
| P42 | Unknown bacteria |  |  |  | | yes |  |  |  |  | |  | yes |  |  |  |  | yes |  |  | |  |  |
| P43 | Unknown bacteria |  |  |  | | yes |  |  |  |  | |  | yes |  |  |  |  | yes |  |  | |  |  |
| P44 | Unknown bacteria |  |  |  | | yes |  |  |  |  | |  | yes |  |  |  |  | yes |  |  | |  |  |
| P45 | Unknown bacteria |  |  |  | | yes |  |  |  |  | |  | yes |  |  |  |  | yes |  |  | |  |  |
| P46 | Unknown bacteria |  |  |  | | yes |  |  |  |  | |  | yes |  |  |  |  | yes |  |  | |  |  |
| P47 | Unknown bacteria |  |  |  | |  |  |  |  |  | |  | yes |  |  |  |  | yes |  |  | |  |  |
| P48 |  | Aspergillus genus |  |  | | yes |  |  |  | yes | |  |  |  |  |  |  |  | yes |  | | yes |  |
| P49 |  | Aspergillus genus |  |  | | yes |  |  |  | yes | |  |  |  |  |  |  |  | yes |  | |  |  |
| P50 |  | Aspergillus genus |  |  | | yes |  |  |  | yes | |  |  |  |  |  |  |  | yes |  | | yes |  |
| P51 | S. aureus, E.aerogen, Enterobacter cloacae complex | Aspergillus genus |  |  | |  | yes |  |  |  | | yes |  |  |  |  |  |  | yes | MRSA, ESBLs | | yes |  |
| P52 | A. baumanii, E. faecium | A. fumigatus, A.flavus | Influenza virus |  | |  | yes |  |  |  | | yes |  |  |  |  |  |  | yes |  | | yes |  |
| P53 |  | A. fumigatus |  |  | | yes |  |  |  | yes | |  |  |  |  |  |  |  | yes |  | |  |  |
| P54 |  | A. fumigatus |  |  | | yes |  |  |  | yes | |  |  |  |  |  |  |  | yes |  | |  |  |
| P55 | K. pneumoniae | A. fumigatus |  |  | | yes |  |  |  | yes | |  |  |  |  |  |  |  | yes | CRKP | | yes |  |
| P56 | K. pneumoniae |  |  |  | | yes |  |  |  | yes | |  |  |  |  |  |  |  | yes | CRKP | |  |  |
| P57 |  | A. flavus |  |  | | yes |  |  |  | yes | |  |  |  |  |  |  |  | yes |  | |  |  |
| P58 |  | A. fumigatus |  |  | | yes |  |  |  | yes | |  |  |  |  |  |  |  | yes |  | | yes |  |
| P59 | P.aeruginosa, A. baumanii, Acinetobacter pittii, S.capitis | A. fumigatus |  | yes | |  | yes |  |  |  | | yes |  |  |  |  |  |  | yes |  | | yes |  |
| P60 | Rickettsial tsutsugamushioi | Mucorales |  | yes | |  | yes |  |  |  | | yes |  |  |  |  |  |  | yes |  | | yes |  |
| P61 | Mycoplasma |  |  |  | | yes |  |  |  |  | |  |  | yes |  |  |  |  | yes |  | |  |  |
| P62 | S.aureus, P.aeruginosa |  |  |  | | yes |  |  |  | yes | |  |  |  |  |  |  |  | yes | MSSA | | yes |  |
| P63 | P.maltophila |  |  |  | | yes |  |  |  | yes | |  |  |  |  |  |  |  | yes |  | |  |  |
| P64 |  | Candida |  |  | |  |  |  | yes | yes | |  |  |  |  |  |  |  | yes |  | | yes |  |
| P65 | Mycoplasma |  |  |  | | yes |  |  |  |  | |  |  | yes |  |  |  |  | yes |  | |  |  |
| P66 | E.cloacae |  |  | yes | |  |  |  |  | yes | |  |  |  |  |  |  |  | yes | ESBLs | |  |  |
| P67 | Actinomyces neuii |  |  | yes | |  |  |  |  | yes | |  |  |  |  |  |  |  | yes |  | |  |  |
| P68 | Rhizopus, P. putida, E.faecium |  |  |  | | yes |  | yes |  | yes | |  |  |  |  |  |  |  | yes |  | |  |  |
| P69 | A. baumanii, S.haemolyticus |  |  | yes | | yes |  |  |  | yes | |  |  |  |  |  |  |  | yes |  | |  |  |
| P70 |  | Unknown fungus |  |  | |  |  |  |  |  | |  |  |  | yes |  |  |  | yes |  | |  |  |
| P71 |  | Unknown fungus |  |  | |  |  |  |  |  | |  |  |  | yes |  |  |  | yes |  | |  |  |
| P72 |  | Unknown fungus |  |  | |  |  |  |  |  | |  |  |  | yes | yes | yes |  | yes |  | |  |  |
| P73 | Unknown bacteria |  |  |  | | yes |  |  |  |  | |  |  |  | yes |  |  |  | yes |  | |  |  |
| P74 | Unknown bacteria |  |  |  | | yes |  |  |  |  | |  | yes |  |  |  |  |  | yes |  | |  |  |
| P75 | **Unknown bacteria** |  |  |  | | yes |  |  |  |  | |  | yes |  |  |  |  |  | yes |  | |  |  |
| P76 |  | Unknown fungus |  |  | |  |  |  |  |  | |  |  |  | yes | yes | yes |  | yes |  | | yes |  |
| P77 |  | Unknown fungus |  |  | |  |  |  |  |  | |  |  |  | yes |  |  |  | yes |  | |  |  |
| P78 | Unknown bacteria |  |  |  | | yes |  |  |  |  | |  |  |  | yes |  |  |  | yes |  | |  |  |
| P79 | Unknown bacteria |  |  |  | | yes |  |  |  |  | |  | yes |  |  |  |  |  | yes |  | |  |  |
| P80 |  | Unknown fungus |  |  | |  |  |  |  |  | |  |  |  | yes |  |  |  | yes |  | |  |  |
| P81 |  | Unknown fungus |  |  | |  |  |  |  |  | |  |  |  | yes |  |  |  | yes |  | | yes |  |

**Abbreviations**: ID, Identity; CRKP, carbapenem-resistant *K. pneumoniae*; ESBLs, extended-spectrumβ-lactamases; MRSA, methicillin-resistant *staphylococcus aureus*; MSSA, methicillin sensitive *staphylococcus aureus*; mNGS, Metagenomic next-generation sequencing; CT, computerized tomography; GM test, Galactomannan; G test, 1-3-β-D glucan test.

Supplementary Table S2. Coefficients of Lasso regression analysis.

| **Variables** | **Coefficients** |
| --- | --- |
| Diarrhea | 1.084419 |
| Cerebral infarction | 0.918399 |
| Ribavirin | 0.032416 |
| Antibiotic therapy | 0.346891 |
| Antifungal therapy | 2.929639 |
| Blood Products | 0 |
| Transfer to ICU | 0.999352 |
| Myocardial injury | 0.390845 |
| Respiratory failure | 0 |
| cTnI | 0 |
| ALB | 0 |
| A/G | 0 |
| Lipase | 0.000234 |
| Amylase | 0 |
| D-Dimer | 0.043898 |
| FDP | 0 |
| Hospitalization stay | 0 |
| Hospitalization expenses | 0 |
| ALB<35.5g | 0 |
| A/G<1.05 | 0.417508 |

**Abbreviations**: ICU, Intensive care unit; ALB, Albumin; A/G, Albumin/globulin ratio; FDP, fibrinogen degradation product.
